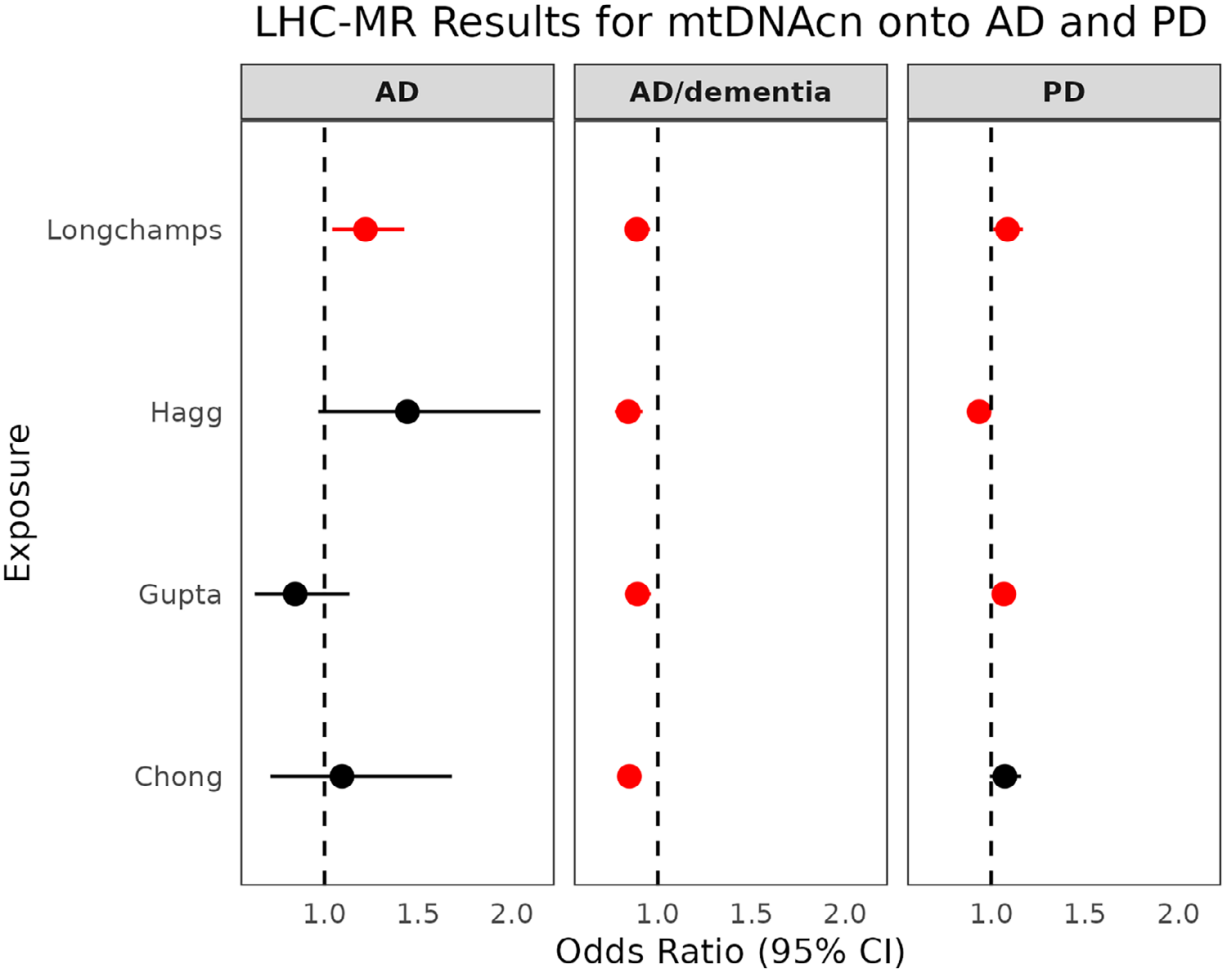# Evaluating the Causal Effect of Mitochondrial Dysfunction on Alzheimer’s and Parkinson’s Disease using Polygenic Risk Scores and Mendelian Randomization

**DOI:** 10.1002/alz70861_108688

**Published:** 2025-12-23

**Authors:** Aadrita Chatterjee, Brian D Alvarez, Rakshya U Sharma, Heather M Wilkins, Russell H Swerdlow, Kristine Yaffe, Shea J Andrews

**Affiliations:** ^1^ University of California, San Francisco, San Francisco, CA USA; ^2^ Icahn Mount Sinai, New York, NY USA; ^3^ University of Kansas Medical Center, Kansas City, KS USA; ^4^ San Francisco Veterans Affairs Health Care System, San Francisco, CA USA; ^5^ Department of Psychiatry, Neurology, and Epidemiology and Biostatistics University of California San Francisco School of Medicine, San Francisco, CA USA; ^6^ Department of Psychiatry and Behavioral Sciences, University of California ‐ San Francisco, San Francisco, CA USA

## Abstract

**Background:**

Mitochondrial DNA copy number (mtDNAcn) quantifies the number of mitochondria genomes per nucleated cell, with lower mtDNAcn linked to increased Alzheimer’s disease (AD) and Parkinson’s disease (PD) risk. Multiple approaches exist to quantify mtDNAcn including quantitative PCR, microarray‐based intensity estimates, whole exome sequencing, and whole genome sequencing—each offering different resolution, cost, and sensitivity. In this comprehensive analysis, we utilized genome‐wide summary statistics (GWAS) for four different measures of mtDNAcn to combine Polygenic Risk Scores (PRS) and two‐sample Mendelian randomization (MR). This approach allowed us to examine both the association and potential causal role of mtDNAcn in AD and PD.

**Method:**

We used GWAS for mtDNAcn, AD/AD‐dementia and PD. In the Alzheimer’s Disease Genetics Consortium (*N* =27,383; male= 39.4%; age=75 [69‐81]; cases=37.8%; European=80%), ancestry‐normalized PRS were generated using all four mtDNAcn GWAS (PRS‐CS‐auto, 1000 Genomes European reference panel, HapMap3 SNPs, excluding *APOE* region). Logistic regression assessed the association between mtDNAcn PRS and AD status, adjusting for age, sex, *APOE*‐ε4 status and population stratification. For MR, Inverse Variance Weighted was the primary method with sensitivity analyses. We also applied Latent Heritable Confounder MR (LHC‐MR) to estimate the causal effect of mtDNAcn on AD while accounting for unmeasured confounding.

**Result:**

PRS analysis showed one of four mtDNAcn datasets significantly associated with AD. Using LHC‐MR, higher genetically predicted mtDNAcn was causally associated with AD/dementia using all four mtDNAcn datasets (Figure 1). Furthermore, using LHC‐MR three mtDNAcn datasets were causally associated with PD (Figure 1). However, univariate MR showed no significant causal effect of mtDNAcn on AD, AD/dementia, or PD.

**Conclusion:**

Higher blood‐based mtDNAcn was causally associated with reduced risk of AD/dementia, with limited evidence to suggest a bidirectional effect. There was also limited evidence to support a causal effect of mtDNAcn onto PD. Discrepancies between univariate and LHC‐MR results may be explained by sample overlap issues in univariate MR as well as by the increased power and the inclusion of a latent confounder in LHC‐MR.